# Three dimensional structures of putative, primitive proteins to investigate the origin of homochirality

**DOI:** 10.1038/s41598-019-48134-z

**Published:** 2019-08-12

**Authors:** Akifumi Oda, Tomoki Nakayoshi, Koichi Kato, Shuichi Fukuyoshi, Eiji Kurimoto

**Affiliations:** 1 Meijo University, Faculty of Pharmacy, Nagoya, 468-8503 Japan; 2 Osaka University, Institute for Protein Research, Suita, 565-0871 Japan; 3 Kanazawa University, Faculty of Pharmacy, Institute of Medical, Pharmaceutical and Health Sciences, Kanazawa, 920-1192 Japan; 4 Kinjo Gakuin University, Faculty of Pharmacy, Nagoya, 463-8521 Japan

**Keywords:** Protein structure predictions, Origin of life

## Abstract

Primitive proteins are likely to have been constructed from non-enzymatically generated amino acids, due to the weak enzymatic activities of primitive biomolecules such as ribozymes. On the other hand, almost all present proteins are constructed only from l-amino acids. Therefore, there must have been a mechanism early in the origins of life that selected for one of the optical isomers of amino acids. In this study, we used molecular dynamics simulations to predict the three-dimensional structures of the putative primitive proteins constructed only from glycine, alanine, aspartic acid, and valine ([GADV]-peptides). The [GADV]-peptides were generated computationally at random from l-amino acids (l-[GADV]-peptides) and from both l- and d-amino acids (dl-[GADV]-peptides). The results indicate that the tendency of secondary structure formation for l-[GADV]-peptides was larger than that for dl-[GADV]-peptides, and l-[GADV]-peptides were more rigid than dl-[GADV]-peptides. These results suggest that the proteins with rigid structure motifs were more prone to have been generated in a primordial soup that included only l-amino acids than a the soup including racemic amino acids. The tendency of the rigid structure motif formation may have played a role in selecting for the homochirality that dominates life on Earth today.

## Introduction

In the Hadean eon (about 4,000 million years ago), the first life was generated on Earth. Although the specific details of the origin of life are unclear, several hypotheses about the components of primitive life have been proposed. For example, in the RNA world hypothesis, the first life was presumed to be constructed from RNA^1^. On the other hand, the protein world hypothesis states that life was considered to originate from proteins^2,3^. The protein world hypothesis is one of the “metabolism-first” scenarios in which self-sustaining chemical reactions are considered to be the ancestor of what we call life. In such hypotheses, primitive biomolecules are presumed to act as catalysts for metabolic reactions. The history of metabolism-first hypotheses dates back to Oparin’s coacervate theory^4^. Various other metabolism-first scenarios have been proposed such as early chemical evolution theories by Haldane^5^, iron-sulfur world^6^, garbage-bag world^7^, and protein world. In the GADV hypothesis, the proteins constructed from glycine (G), alanine (A), aspartic acid (D), and valine (V) ([GADV]-proteins) were the primary components of primitive life^2,3^. In addition, because G, A, D, and V can be synthesized easily from inorganic compounds under primitive Earth conditions without enzymes, [GADV]-proteins are considered the important biomolecules not only for the protein world hypotheses but also for the RNA world hypothesis^8^. Although proteins today include around 20 types of amino acids, many researchers have considered that primitive proteins were constructed from a limited set of amino acids^2,3,8–11^. The [GADV] amino acid set includes both hydrophobic and hydrophilic amino acids, and all four amino acids are coded for by the universal code GNC. This indicates that these four amino acids can generate proteins with various properties by a simplified genetic code.

Recently, we have predicted the three-dimensional (3D) structures of randomly generated [GADV]-proteins by using computational chemical methods^12^. The results of these studies suggest that even short and randomly generated peptide chains, including only G, A, D and V, can form secondary structures that might act as proteins. On the other hand, peptides used in those studies include only l-amino acids, but d-amino acids were not considered. If the amino acids were constructed by non-enzymatic reactions on the primitive Earth in the Hadean eon, the amino acids must have existed in a racemic form. Even researchers who advocate for an RNA world hypothesis agree that non-enzymatically generated amino acids are important components of life because of the weak enzymatic activity of ribozymes. Miller reported that several racemic amino acids were constructed under the putative primitive Earth condition^13^. After the Miller experiment, several researchers have reported that various amino acids can be generated non-enzymatically in a putative primitive Earth environment^14–21^. Although the Miller experiment was performed in a strongly reducing atmosphere, amino acids also were generated under a weakly reducing atmosphere^14^. In addition, amino acids generated in hydrothermal vents^15–18^ and in space^19–21^ have been found. Thus, many researchers have considered that non-enzymatically constructed amino acids likely existed on the primitive Earth and are the important components of primitive life. Because non-enzymatically generated amino acids reported previously all were racemic^13–21^, both l- and d-form of amino acids should be used in the structural predictions of the primitive proteins.

In the proteins in extant life, amino acid residues are almost always in the l-form. Although stereoinversion of the amino acid residues have been found in tissues of aged individuals^22^, wild type proteins are almost always constructed from l-amino acids. To date, various hypotheses have been proposed to explain the origin of homochirality of amino acid residues^23–29^. Due to non-enzymatic synthesis, racemic amino acids are generated. Therefore, the inequality in the abundance ratio between l- and d-amino acids was caused at the earliest stages in the origin of life, and the chiral bias was amplified during the course of evolution. Several experimental and computational results have been reported to explain this chiral bias. For example, an enantiometric excess in meteoritic amino acids^23^, chiral bias from amino acid degradation of circular polarization^24^, from the adsorption on crystal surfaces^25^, and from the parity-violating energy shifts of amino acids^26^ have been reported. Thus, some hypotheses for the amplification of chiral bias have been proposed, such as enantioselective crystallization^27^ and enantioselective peptide formation on water^28^. Furthermore, solubility-based amplification of small enantiometric excess has been reported for amino acids as well as nucleosides^29^. Although these hypotheses explain the physical environments and chemical reactions of amino acids and peptides, biological properties of primitive proteins and peptides were not discussed sufficiently. In particular, the structural biological properties of primitive proteins and peptides constructed from homochiral or racemic amino acids have not been investigated. If the structural properties of proteins constructed from homochiral amino acids were different from those of proteins that include both l- and d-amino acids, the origin of homochirality may be explained by the protein property itself without any special external environment. Similarly, the amplification of chiral bias may be explained by the biological properties of primitive proteins. Therefore, investigations of the structural properties of proteins, including both l- and d-amino acids, are expected to play important roles to clarify only l-amino acids were selected during early life, rather than racemic amino acids. Recently, the structural features of proteins constructed from a mixture of l- and d-amino acid residues have been reported^22,30–37^. Several structural changes have been observed in proteins including d-amino acid residues. For example, the aggregation and precipitation of amyloid β (Aβ), elastin, and α-crystallin have been reported^22^. For Aβ, the formation and collapse of β-sheet structures caused by d-amino acids have been observed using circular dichroic spectra^30,31^ and computational chemistry methods^32^. The effects of d-amino acid residues on 3D structures have been investigated not only for natural proteins and peptides but also for artificial ones. The introduction of d-Ala destabilizes a right-handed α-helical coiled coil construct^33^; however, alternating d, l polypeptides were found in which specific types of local structures were stabilized^34^. For artificial peptides, destabilization of the helix structures depended on the types^35^ and positions^36^ of d-amino acids. Both local changes^37^ and whole structural changes^22,30^ were observed upon the introduction of d-amino acid residues. This pro- and anti-structural effect of l, d mixtures prompted our study to clarify the effects of d-amino acids in tentative primitive proteins.

In this study, 3D structures of [GADV]-peptides constructed from racemic amino acids were predicted using molecular dynamics (MD) simulations. Although short peptides cannot form rigid 3D structures, their structural tendency can be evaluated. Recently, we tested the MD simulations for structural predictions of small proteins. The results revealed that structure formation tendencies of small proteins, such as the secondary structure formation tendency, can be predicted by MD simulations^38^. Therefore, we conducted structural predictions of putative primitive proteins using MD simulations. Using the predicted structures, we then investigated various structural properties, including the frequencies of secondary structure formation and hydrogen bonding frequencies, to clarify the influence of d-amino acids in the primitive proteins.

## Results and Discussion

**Figure 1:**
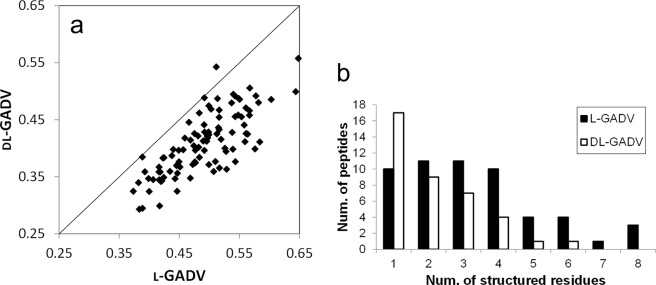
Secondary structures in [GADV] peptides. (**a**) The secondary structure contents. Vertical and horizontal axes indicate the secondary structure contents throughout the last 10 ns trajectories. Each point represents one sequence. The line represents the 1:1 line, where secondary structures are equal for both types of peptides. (**b**) The number of peptides classified by the number of the structured residues. When the residue formed secondary structure at 50% of trajectory, the residue was defined as “structured residue.”

In this study, the peptides that included only l-amino acids are referred to as “l-[GADV] peptides,” and the peptides including both d- and l-amino acids are referred to as the “dl-[GADV] peptides.” We conducted replica exchange MD (REMD) simulations^39^ of 100 l-[GADV] peptides and 100 dl-[GADV] peptides. The amino acid sequences of these peptides are shown in Table S1. In Fig. 1a, the secondary structure contents calculated from MD trajectories were compared between l-[GADV] and dl-[GADV] peptides. The average secondary structure contents were 0.49 and 0.41 for l-[GADV] peptides and dl-[GADV] peptides, respectively. As shown in the figure, more secondary structures were observed for l-[GADV] peptides than dl-[GADV] peptides even though the amino acid sequences were the same. In addition to the secondary structure contents, when residues formed secondary structures at 50% of the trajectory, the residue was defined as a “structured residue.” The number of structured residues indicates the ability to form structures of [GADV]-peptides. The 100 peptides were classified by the number of structured residues (Fig. 1b). Although the maximum number of structured residues was eight for l-[GADV] peptides, at most only six residues can form secondary structure for dl-[GADV] peptides. In addition, 61 dl-[GADV] peptides had no residues that formed secondary structures, whereas 46 l-[GADV] peptides had no structured residues. The average number of structured residues in the 100 peptides were 1.80 and 0.83 for l-[GADV] and dl-[GADV] peptides, respectively. These results indicate that secondary structures are more prone to form in l-[GADV] peptides than in dl-[GADV] peptides. Because the amino acid sequences were the same for l-[GADV] peptides and dl-[GADV] peptides, these results can be explained by diastereomerism. That is, the inclusion of d-amino acids decreases the ability of secondary structure formation in [GADV]-peptides.

When the helix and β structures were analyzed separately, l-[GADV] peptides were more prone to form a helix rather than a β structure (1.76 vs. 0.04). On the other hand, the occurrence of helical and β residues were approximately the same for dl-[GADV] peptides (0.42 vs. 0.41). As in the previous paper^12^, β turns were observed as β structures, and sheet structures were not detected. Of the 200 l-[GADV] and dl-[GADV] peptides, only one peptide included three consecutive β residues (d-Asp12-d-Val13-l-Val14 in peptide 58 of dl-[GADV] peptides), and these three consecutive residues all formed a β turn. In the three residues, alternation between d- and l-amino acids existed (d-Val13-l-Val14). The two consecutive β residues were found in seven dl-[GADV] peptides. In six of the seven dl-[GADV] peptides (including two consecutive β residues), d- and l-forms were bound in consecutive β structures. On the other hand, no l-[GADV] peptides included two consecutive β structures. These results indicate that the bond between d- and l-amino acids is prone to form β structure. However, because only three β residues were found in dl-[GADV] peptides, β structures were not sufficient to explain the rigid protein structure formations. Therefore, although the number of β residues was larger in dl-[GADV] peptides than in l-[GADV] peptides, l-[GADV] peptides seemed more prone to form rigid proteins. As mentioned above, the average number of helix residues for l-[GADV] peptides was more than four times that for dl-[GADV] peptides. By contrast, β-sheet structures were not found in all l-[GADV] and dl-[GADV] peptides. The average β-sheet contents for l-[GADV] and dl-[GADV] peptides were 0.01 and 0.02, respectively. Although occasional exceptions were observed (such as chignolin^40^), helices play an important role in structure formation of the small protein. Because the sequence lengths of the non-enzymatically generated primitive proteins were short, the l-[GADV] peptides that more easily form helix structures appeared to have an advantage in forming proteins under the primitive Earth environment compared to dl-[GADV] peptides.

**Figure 2:**
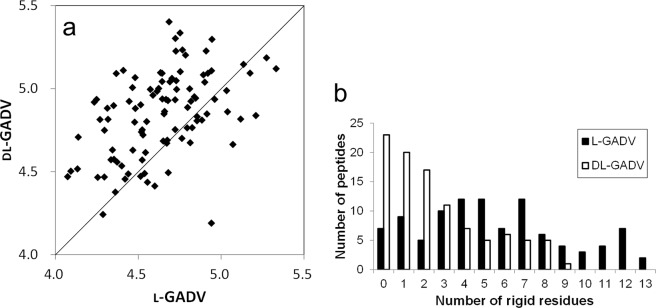
Rigid residues in [GADV] peptides. (**a**) Average RMSF of Cα. Vertical and horizontal axes indicate the average RMSFs of Cα throughout the last 10 ns trajectories. Each point represents one sequence. The line represents the 1:1 line, where RMSF are equal for both types of peptides. (**b**) The number of peptides classified by the number of the rigid residues. The residue in which RMSF of Cα was smaller than 4.0 Å was defined as rigid residue.

As shown in Fig. 1, more secondary structures were observed in l-[GADV] peptides than in dl-[GADV] peptides. However, the concept of secondary structure was defined from the analytical results of existing proteins. Thus, proteins that included atypical amino acids, such as d-amino acids, might form atypical secondary structures that cannot be detected by DSSP. Because the rigidity of the amino acid chain plays an important role in protein function, helix, and β structures, as well as atypical secondary structures, may act as functional motifs of proteins. The structure of the catalytic site plays an important role not only in enzymes but also in simpler molecular catalysts^41,42^. When a molecule acts as a catalyst, the catalytic moiety (“active site”) must be located near the reaction point of the reactant and an appropriate reactant complex must be formed. The appropriate structure of the reactant complex facilitates a chemical reaction^43,44^. Because the locations of catalytic moieties have to be fixed, a certain level of rigidity plays an important role in primitive proteins that can act as primitive enzymes. Therefore, to investigate the rigidity of [GADV]-peptides, we calculated the RMSF of Cα atoms, which indicates the structural flexibility of Cα atoms. Smaller RMSF values indicate more rigid structures of the atoms. In Fig. 2a, the average RMSFs of Cα atoms were compared between l-[GADV] and dl-[GADV] peptides. The average of the average RMSFs were 4.64 and 4.84 for l-[GADV] peptides and dl-[GADV] peptides, respectively. As shown in the figure, l-[GADV] peptides were more rigid than dl-[GADV] peptides even though the amino acid sequences were the same. In addition to the average RMSFs, the numbers of rigid residues were evaluated. In these calculations, residues with RMSF < 4 Å were defined as “rigid” residues. The numbers of the peptides classified by the number of the rigid residues were illustrated in Fig. 2b. Peptides that included 13 rigid residues were found in l-[GADV] peptides, although at most, nine rigid residues were observed in dl-[GADV] peptides. Only seven l-[GADV] peptides had no rigid residues, but 23 dl-[GADV] peptides had no rigid residues. The average number of rigid residues was 5.51 and 2.60 for l-[GADV] peptides and dl-[GADV] peptides, respectively. In addition, average RMSFs of all residues (20 residues × 100 peptides = 2000 residues) were 4.64 Å and 4.84 Å for l-[GADV] peptides and dl-[GADV] peptides, respectively. These results indicate that the l-[GADV] peptides are more rigid than dl-[GADV] peptides. In other words, the structure of peptides becomes flexible if d-amino acids are included. Therefore, l-[GADV] peptides tend to form normal secondary structures, such as α helix and β structures, as well as other ordered structures, in comparison with dl-[GADV] peptides. These results suggest that l-[GADV] peptides are more “protein-like” than dl-[GADV] peptides.

**Figure 3:**
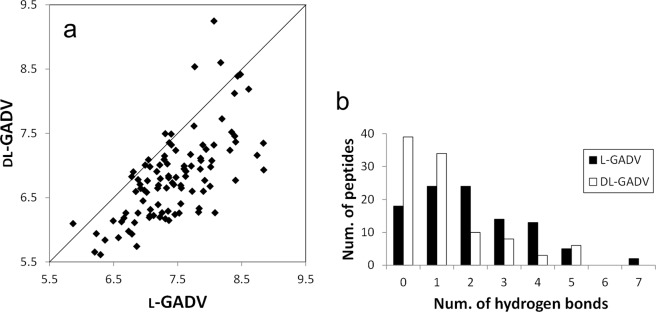
Numbers of the hydrogen bonds. (**a**) Average number of the hydrogen bonds. Vertical and horizontal axes indicate the average numbers of hydrogen bonds throughout the last 10 ns trajectories. Each point represents one sequence. The line represents the 1:1 line, where numbers of bonds are equal for both types of peptides. (**b**) The number of peptides classified by the number of the frequently observed hydrogen bonds. Hydrogen bonds that were observed in 30% of the snapshots in the last 10 ns trajectories were defined as frequently observed hydrogen bonds.

In Fig. 3a, the trajectory average of the numbers of hydrogen bonds in each peptide is compared between l-[GADV] and dl-[GADV] peptides. Even though the amino acid sequences were the same, more hydrogen bonds were observed for l-[GADV] peptides than dl-[GADV] peptides (Fig. 3a). The average for the 100 trajectory averages of the numbers of hydrogen bonds were 7.44 and 6.82 for l-[GADV] peptides and dl-[GADV] peptides, respectively. These results suggest that hydrogen bonds become difficult to form if d-amino acids are included in the tentative primitive proteins. Because the amino acid sequences were the same, the correlation coefficient of the average numbers of hydrogen bonds between l-[GADV] peptides and dl-[GADV] peptides was high (0.670). On the other hand, despite having the same sequences, the number of hydrogen bonds decreased by including d-amino acids. The decreased number of hydrogen bonds may affect the difficulty of rigid structure formation of tentative primitive [GADV]-proteins, including d-amino acids.

In Fig. 3a, all snapshots in the last 10 ns trajectories were used for analyses. Therefore, even rarely observed hydrogen bond that were found in only a few snapshots were counted. However, continuously formed hydrogen bonds play more important roles in protein structure formation than rarely observed hydrogen bonds. Therefore, frequently observed hydrogen bonds were analyzed separately. Hydrogen bonds that were observed in 30% of the snapshots in the last 10 ns trajectories were defined as “frequently observed hydrogen bonds.” The number of frequently observed hydrogen bonds is expected to act as an indicator of structure formation tendency. In Fig. 3b, the numbers of the peptides classified by the number of the frequently observed hydrogen bonds were illustrated. For l-[GADV] peptides, not only the average number of hydrogen bonds shown in Fig. 3a but also the numbers of the frequently observed hydrogen bonds shown in Fig. 3b were larger than for dl-[GADV] peptides. There were 18 and 39 peptides in which there were no frequently observed hydrogen bonds for l-[GADV] peptides and dl-[GADV] peptides, respectively (Fig. 3b). In fact, more than 70% of dl-[GADV] peptides included only one or zero frequently observed hydrogen bonds. The average number of the frequently observed hydrogen bonds was 2.05 and 1.20 for l-[GADV] peptides and dl-[GADV] peptides, respectively. These results indicate that the important hydrogen bonds, which are formed continuously to maintain the rigid protein structure, are more difficult to form in dl-[GADV] peptides.

**Figure 4:**
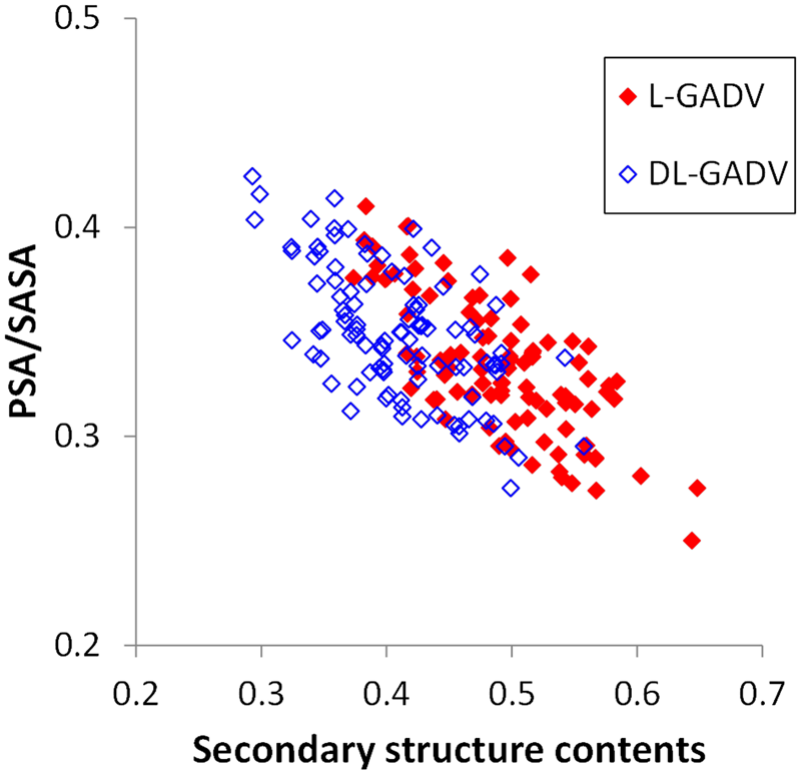
Relationship between PSA/SASA and secondary structure contents. The ratio between the trajectory averages of PSA and SASA was defined as PSA/SASA. The secondary structure content is the ratio of the number of structured residues to the number of all residues, and it is compatible with the secondary structure content obtained by experiments such as circular dichroic spectrum. Each point represents one peptide, and there are 100 red dots and 100 blue dots corresponding to the 100 l-[GADV] and 100 dl-[GADV] peptides, respectively.

For all snapshots of peptides, PSA and SASA were calculated. PSA/SASA was negatively correlated with secondary structure content (Fig. 4). The correlation coefficients were −0.721 and −0.616 for l-[GADV] peptides and dl-[GADV] peptides, respectively. This result suggests that the more hydrophobic peptides can form more secondary structures, and the hydrophobic effects play important roles in structure formations of proteins. Although PSA/SASA values of dl-[GADV] peptides were slightly higher than for l-[GADV] peptides, the difference was not substantial. The average PSA/SASA for the 100 peptides was 0.333 and 0.349 for l-[GADV] peptides and dl-[GADV] peptides, respectively. On the other hand, the difference in the average value of secondary structure contents was large (0.491 vs. 0.409 for l-[GADV] peptides and dl-[GADV] peptides, respectively). This result indicates that the ability of secondary structure formation for l-[GADV] peptides is superior to that for dl-[GADV] peptides, even if the water solubilities are similar. Because primitive life likely originated in water environments, l-[GADV] peptides appear to be more suitable for primitive proteins, because of a fine balance between water solubility and secondary structure formation ability. Especially for helix contents, the average value of l-[GADV] peptides was larger than that of dl-[GADV] peptides (0.253 for l-[GADV] peptides and 0.163 for dl-[GADV] peptides). Therefore, l-[GADV] peptides seem to be more prone to form helical proteins compared to dl-[GADV] peptides. This study compared l-amino acids and the racemic mixture. The AMBER force field parameters^45,46^ for the d-amino acids used in this study are the same as those for the l-amino acids — chirality being enforced by the relative starting positions of atoms^47^. Therefore, by definition, there is no difference between all-L and all-D systems in these MD simulations. The point of this study was not to justify the choice of l- over d-amino acids, but to investigate homochiral vs. racemic amino acids. Therefore, our results do not explain an all-L world, specifically, but a homochiral world. The results of the present study indicate that homochiral [GADV]-peptides are more prone to forming stable structures compared to racemic [GADV]-peptides.

## Conclusion

In this study, the 3D structures were investigated with REMD simulations for two types of [GADV]-peptides, one constructed only from l-amino acids and the other that included both l- and d-forms. The results showed that l-[GADV] peptides are more prone to form the rigid and well-ordered structures than dl-[GADV] peptides. Therefore, the “primordial soup” that excluded d-amino acids seems to be more appropriate for an environment favoring the origin of proteins than a soup that included both l- and d-amino acids in the GADV hypothesis. This study compared l-amino acids and the racemic mixture, not l- and d-amino acids. In fact, Towse *et al*. reported that artificial peptides constructed from only d-amino acids can form appropriate conformations as well as peptides consisting of only l-amino acids^48^. Our results suggest that the homochiral primordial soup, which included only l- or d-amino acids, is more preferable as the birthplace of primitive proteins than racemic soup for [GADV]-peptides.

As mentioned above, although the non-enzymatically synthesized amino acids are racemic, many researchers have considered that the chiral bias was caused in the primordial soup by other reasons, such as cosmic rays, parity-violating energy shifts, or mere chance. Because extant proteins are almost all constructed from l-amino acids, the homochirality must have been achieved in the very earliest stages of the origin of life. Our results suggest that the primitive [GADV]-proteins were more prone to originate in a chiral-biased primordial soup than in a racemic soup. In addition, in biological systems, rigid, and structured [GADV]-proteins are more prone to be constructed when only l-amino acids are generated. Because protein production is the key for reproduction, biological systems that can generate proteins more easily seem to reproduce more easily. That is, 3D structure formation of proteins may act as a selection pressure, and the biological system that can generate only l-amino acids may be the one selected. By contrast, systems that generated racemic amino acids were inferior, in terms of [GADV]-protein formation ability, and the inferiority of the protein production may cause delays in the origin of life in the racemic environment. Thus, a racemic system may have been outcompeted, in favor of a chiral-biased system. As future development of this study, structural predictions and hydrogen bonding analyses using the resulting structures of this study in the explicit solvent are needed. To investigate protein quaternary structure, such as amyloid-forming ability, multi-chain simulations should be carried out. The structural formation tendencies of other combinations of amino acids than [GADV]—especially amino acid sets including non-proteogenic amino acids like isovaline and β-alanine—are currently being evaluated. The results of this study indicate that peptides constructed only by l-amino acids are more prone to evolve into complex proteins, which may be one reason for the origin of homochirality of life.

## Methods

### [GADV]-peptides

Similar to our previous study^12^, we used a sequence length of 20 for the [GADV]-peptides. In the peptides, amino acid sequences were determined randomly, and each residue was stereoinverted randomly at a 50% probability. The occurrence rates of G, A, D, and V in the peptides were all 25% (equal rates). A total of 100 sequences were generated for the test. In addition, the peptides including all l-amino acids were also constructed for comparison. To compare between l-[GADV] and dl-[GADV] peptides, we generated the same 100 sequences for both peptide types. For the test peptides, initial 3D structures were generated by the tleap module of AmberTools in the AMBER12 program^49^. To avoid bias from initial structures, linear structures were used for the initial of MD simulations.

### Molecular Simulations

First, structural minimizations were performed for the initial linear structures. The maximum number of minimization cycles was set to 2000. After minimization, MD simulations were conducted using the minimized structures. For the structural predictions of [GADV]-peptides, we used REMD^39^. The calculations were conducted using AMBER12^49^. Because we already tested the applicability of the force field parameters for d-amino acids^47^, the AMBER ff12SB force field was used for both l- and d-amino acids. The cutoff distance for non-bonding terms was 999 Å, indicating that no cutoff calculations were performed. Water solvent was treated as a generalized Born implicit solvent using a GBneck 2 model (igb = 8)^50^ with an mbondi3 atomic radius, and the salt concentration was set at 0.1 M. A total of 50 ns REMD simulations were carried out with a 2 fs time step. Sixteen replicas were used for calculations, and the replica exchange was performed at each 500 steps. The temperatures of replicas were set at 269.7 K, 284.4 K, 300.0 K, 316.4 K, 333.8 K, 352.0 K, 371.3 K, 391.7 K, 413.1 K, 435.7 K, 459.6 K, 484.8 K, 511.3 K, 539.3 K, 568.8 K, and 600.0 K. Before REMD simulations, we performed 200 ps normal MD simulation for each replica to equilibrate the system at each temperature. For all MD simulations, we enforced restrictions to avoid chiral inversions at high temperatures. The interatomic distances of covalent bonds including hydrogen atoms, e.g., hydrocarbon C-H, hydroxyl O-H, and amide N-H, were constrained according to the SHAKE method. Previously, we evaluated the effectiveness of MD simulations for structural predictions of short proteins^38,51^. The results of benchmark studies indicate that the REMD simulation used in this study can be used for secondary structure predictions of small proteins.

### Analyses

For the data analyses, the final 10 ns trajectories from a total of 50 ns simulations were used. Because the 3D structures were stored every 1 ps, 10,000 snapshots were included in the 10 ns trajectory. The secondary structures of all snapshots were analyzed, and the secondary structure contents were calculated. The assignments of the secondary structures of proteins were performed with DSSP^52^. For the DSSP results, parallel and anti-parallel sheets and turns were defined as “β structures,” and α, 3–10, and π helixes were considered as “helix.” The definitions of these secondary structures are identical to those used by refs^12^,^32,]^^53^. In addition, root mean square fluctuations (RMSF) of Cα atoms were calculated to evaluate the structural flexibilities of [GADV]-peptides. Because the concept of secondary structure is based on the 3D structures of currently existing proteins, primitive proteins might form unique secondary structures even when they can form well-ordered structures. For example, α strands and β helices were found in the alternating d, l polypeptides^34^. To detect ordered structures other than general secondary structures, RMSF values were calculated. Because the RMSF value indicates the flexibilities of peptides, the rigid portion of the peptides can be detected by forming ordered structures. In addition, the number of hydrogen bonds observed in each snapshot also was counted. The number of intramolecular hydrogen bonds is related to the 3D structure formation. The default settings of the cpptraj module of AmberTools were used to detect hydrogen bonds for all 10,000 snapshots of the last 10 ns REMD trajectory, and the average number of hydrogen bonds across all 10,000 snapshots was defined as the “trajectory average.” The trajectory average of the numbers of the hydrogen bonds was calculated for each peptide. Furthermore, the solvent accessible surface area (SASA) and the polar surface area (PSA) of the peptide was calculated using each snapshot. SASA is the molecular surface area that can be accessed by the probe sphere with a 1.4 Å radius. PSA is the surface area of nitrogen and oxygen exposed to solvent water and is used generally as an indicator of hydrophilicity^54^. The RMSF calculations and assignments of hydrogen bonds were conducted with the cpptraj module of AmberTools. SASA and PSA were calculated with the dms program^55,56^.

## Supplementary information

Supplementary information

## Data Availability

The data that support the findings of this study are available from the corresponding author upon reasonable request.
