# Supplementary material for: Three dimensional structures of putative, primitive proteins to investigate the origin of homochirality
Source: Sci Rep. 2019 Aug 12;9:11594. doi: 10.1038/s41598-019-48134-z (PMC6690948; doi:10.1038/s41598-019-48134-z)
Supplement: Supplementary file 1 — Supplementary information [file 41598_2019_48134_MOESM1_ESM.docx]

**Three dimensional structures of putative, primitive proteins to investigate the origin of homochirality**

Akifumi Oda^1,2,3,*^, Tomoki Nakayoshi^1,3^, Koichi Kato^1,4^, Shuichi Fukuyoshi^3^, Eiji Kurimoto^1^

^1^Meijo University, Faculty of Pharmacy, Nagoya, 468-8503, Japan

^2^Osaka University, Institute for Protein Research, Suita, 565-0871, Japan

^3^Kanazawa University, Faculty of Pharmacy, Institute of Medical, Pharmaceutical and Health Sciences, Kanazawa, 920-1192, Japan

^4^Kinjo Gakuin University, Faculty of Pharmacy, Nagoya, 463-8521, Japan

*oda@meijo-u.ac.jp

Table S1 Amino acid sequences of GADV peptides used in this study.

| No. | Sequence ^a)^ | No. | Sequence ^a)^ | No. | Sequence ^a)^ |
| --- | --- | --- | --- | --- | --- |
| 1 | VGdGdagdgvvgGGAVVaAg | 35 | gDDGvdaGgvagdgDaaAAG | 69 | GgVvGgaaGgaDAgVVadag |
| 2 | dDdvgaGdADdAvDGGDDdA | 36 | vaVDGGgGvdaavVdDvAaa | 70 | DVvGgdAvAvaDAvVaddaD |
| 3 | GAAgAvvgAaGdadgAgVAA | 37 | dGDDVaDVgaVdgVAVvAdV | 71 | vGDaAGGGgadDvVVDadaa |
| 4 | AAdvVAaAAvdvAVgVVvGa | 38 | AADAvadgvDADgVvAAaGA | 72 | DDgGAGdvVAGagvAGVdGG |
| 5 | vvAvgavDGavVvGDaAGgd | 39 | gAaVdvAavVgadGggGdGG | 73 | aaagGvaVdVVdDVVadvVa |
| 6 | gAGvaavvdAdAagAGGgvD | 40 | AAAdAGvVgDDvVaAGGdgG | 74 | VavAVdVAGdgaAVGvDdaA |
| 7 | gDDavavAdaVVaDGDvGDD | 41 | VADGGgDVvgdvdVDAVdAd | 75 | dgDgVaaDaDgVGgdgVVAA |
| 8 | GVDddvdGAdGGaAagaGaA | 42 | vvggvDaAdAAgaGgDVggd | 76 | vDAaGVvVGvDvdvDGdGgg |
| 9 | ADaAdGdvAvaaAagdaaAg | 43 | GDdGgvaDgdGvvDDDAgaA | 77 | DvvGGgdDVdavdagdVAvA |
| 10 | gAgvvgGaAvGgagggaDDv | 44 | AagagvVddVDaDAdVAggv | 78 | VdvVgDDAVVGVvvgggADV |
| 11 | AVGvvavVGgdaGdDddGag | 45 | ADDVAvdgaaGgADaDgAGv | 79 | GgADGVvGvDAGDgdgADDG |
| 12 | DddAddgvvgVDaGdaAVdd | 46 | DVDdaDDDVDvggAvgDADV | 80 | AdgdGdVavDDdAGgdaDAd |
| 13 | AVvVDvvaAaVvGaggdGDv | 47 | VvAdaaGDaavdVAVdvdGG | 81 | GvvvgvagGVAvaddddGdV |
| 14 | gvdaAgaaVGvAvVgaDDDA | 48 | VaAgGdvdvGvAGvvaVDDg | 82 | VGddAddavgDgGAVDVava |
| 15 | daGvAgAaGgAaVAGagDAg | 49 | DVggGDVGdAgADaAdvaDg | 83 | ddvDvGdvAVAdGVDdGAAA |
| 16 | aVAaDAAVDdGGVgGVVvvG | 50 | ADdaGvGvAggaggdVGGvv | 84 | dGadVdDDAaDAvGaDDDgG |
| 17 | AGadaDVagDgvvADaVGAV | 51 | daVgaGDaVDDAGADggVdV | 85 | GddVAaaaVAdVdDAGvAGa |
| 18 | gDvGdDVvgDdDGgdvAGAD | 52 | DdGvaGAvdADvagdvavgv | 86 | dGGvVVvvVVagAaggdGgg |
| 19 | DAvaGGadaAgDAaaAdAdA | 53 | dVgvGggDDaddDdAaaGvg | 87 | VGvaVdGADdDDaddadvdD |
| 20 | VDGVVgVdavagvaAaAAAa | 54 | daaVdAvdGGdVVAADvgVG | 88 | DagVddVvaddGDdGvgDDa |
| 21 | AagagDGdgadgaaDVdVaD | 55 | GDVavVdvaAdaVdVagavV | 89 | gDVAVVAAvaaADAAdGvvA |
| 22 | VgGVGGVvgaGDAVGdaddg | 56 | DVaaVdaDvVGdvdDAadva | 90 | aavavdgaGGaAGVgadVaV |
| 23 | dVaaGaGvdgvgGgVDdGVg | 57 | aAVDaDggDddGVaGDvAdA | 91 | gaAVgGgVvdVGDagADadV |
| 24 | DvGAVDaaavvgDGgvdGAD | 58 | gaVDAggdDavGVaGAvAVV | 92 | gVavavaGGgGGAvDdgvgV |
| 25 | dDVgvDdADVVdAADVAgVa | 59 | GAGDdAAvVdddvVGggdVa | 93 | DdvDVgaddVaVAGVgaVgG |
| 26 | AvaVGDaaDDdvvvAAavvA | 60 | VAGgAaaVaDAaVadaDAdD | 94 | GagADvvvGAVgVgAvaDGv |
| 27 | vdgdAADaAGadvgdaGAVV | 61 | dgVavDvAaVGDVaVGaAdV | 95 | VAaAGgdDdgGGDgDaVvDg |
| 28 | VgvDAvVGadAADDvGDADV | 62 | GAgdgVGVAaadDGaadgDv | 96 | VvaavGgADGAvDGgaGDvV |
| 29 | AvavAvaAGDDvgdDgavdV | 63 | DDGggVddVVdVddVaAdVa | 97 | ADgGGGGADadVVDDgvAdG |
| 30 | GggadDvVdaVGgGgAvVAG | 64 | GdDagDVDgvgGaAAgadvD | 98 | gaGadGAvvadddaVGaADd |
| 31 | AvAgdGVAVDDGVGAaDvdV | 65 | DadDaGdavgvDvVdAAavv | 99 | VadaDdVdgDagVVadVAAG |
| 32 | DAagVAagAavVVVaGaaDg | 66 | vgGadgDDAvGADDGavaDD | 100 | AvgVdVDVadddvVDaDGgV |
| 33 | aDDvDdgAdDavdagvDaDG | 67 | GvADADgAAVDGGgvgGvGV |  |  |
| 34 | daVaagaGVGaGaAavgagG | 68 | ADVdgAGDgGddGgvdGDaa |  |  |

^a)^ Lower-case residues are d-form.
